# Remote Ischemic Preconditioning for Prevention of Acute Kidney Injury in Patients Undergoing On-Pump Cardiac Surgery: A Systematic Review and Meta-Analysis

**DOI:** 10.1097/MD.0000000000003465

**Published:** 2016-09-16

**Authors:** Yabing Zhang, Xiyang Zhang, Dongmei Chi, Siyang Wang, Hua Wei, Hong Yu, Qian Li, Bin Liu

**Affiliations:** From the Department of Anesthesiology, West China Hospital of Sichuan University, Chengdu, Sichuan (YZ, XZ, DC, SW, HY, QL, BL), and Department of Anesthesiology, First Affiliated Hospital of Zhengzhou University, Zhengzhou (HW), China.

## Abstract

Remote ischemic preconditioning (RIPC) may attenuate acute kidney injury (AKI). However, results of studies evaluating the effect of RIPC on AKI after cardiac surgery have been controversial and contradictory.

The aim of this meta-analysis is to examine the association between RIPC and AKI after on-pump cardiac surgery.

The authors searched relevant studies in PubMed, EMBASE, and the Cochrane Library through December 2015.

We considered for inclusion all randomized controlled trials that the role of RIPC in reducing AKI and renal replacement therapy (RRT) among patients underwent on-pump cardiac surgical procedures.

We collected the data on AKI, initiation of RRT, serum creatinine (sCr) levels, and in-hospital mortality. Random- and fixed-effect models were used for pooling data.

Nineteen trials including 5100 patients were included. The results of this meta-analysis showed a significant benefit of RIPC for reducing the incidence of AKI after cardiac interventions (odds ratio [OR] = 0.84; 95% confidence interval [CI], 0.73–0.98; *P* = 0.02). No significant difference was found in the incidence of RRT between RIPC and control (OR, 0.76, 95% CI, 0.46–1.24; *P* = 0.36). In addition, compared with standard medical care, RIPC showed no significant difference in postoperative sCr (IV 0.07; 95% CI, −0.03 to 0.16; *P* = 0.20; postoperative day 1; IV 0.00; 95% CI, −0.08 to 0.09; *P* = 0.92; postoperative day 2; IV 0.04; 95% CI, −0.05 to 0.12; *P* = 0.39; postoperative day 3), and in-hospital mortality (OR, 1.21, 95% CI, 0.64–2.30; *P* = 0.56).

According to the results from present meta-analysis, RIPC was associated with a significant reduction AKI after on-pump cardiac surgery but incidence of RRT, postoperative sCr, and in-hospital mortality. Further high-quality randomized controlled trials and experimental researches comparing RIPC are desirable.

## INTRODUCTION

Up to 30% patients developed acute kidney injury (AKI) after cardiac surgery and approximately 1% to 2% of all the patients require renal replacement therapy (RRT).^1,2^ The development of AKI following cardiac surgery is associated with substantial morbidity and mortality, as well as prolonged intensive care unit and hospital stays.^3,4^ The pathophysiology of AKI following cardiac surgery is complex and multifactorial. During cardiopulmonary bypass (CPB), a variety of insults participated the development of tubular injury, including systemic inflammatory response, ischemia–reperfusion (IR) injury, macroscopic and microscopic emboli, exposing blood to nonphysiologic surfaces, significant hemodynamic changes, and exposure to contrast media.^5–7^

Although numerous clinical trials of prophylactic interventions have been used, most of the interventions to prevent AKI after cardiac surgery are not supported definitively by evidence, and some have even proved harmful.^8–10^

Remote ischemic preconditioning (RIPC) is a phenomenon that brief intermittent periods of IR of a distant organ or tissue provide protection to distant organs from subsequent episode of lethal IR.^11,12^ The mechanisms underlying RIPC are incompletely understood. RIPC might preserve kidney function in patients undergoing cardiac and vascular interventions through restraining production of oxygen-free radicals and attenuating the inflammatory cascade response involved in pathogenesis of AKI.^13–15^

The randomized controlled trials (RCTs) concerning the renal protective effect of RIPC in patients undergoing cardiovascular surgery during CPB were inconsistent, and the results remained controversial and contradictory. In this study, we conducted a meta-analysis including complete results from recently published RCT to investigate whether an RIPC protocol prevents AKI after on-pump cardiac surgery. There was no registered protocol.

## METHODS

### Search Strategy

The systematic review was performed in accordance with PRISMA (Preferred Reporting Items for Systematic reviews and Meta-Analyses) guidelines.^16^ Ethical approval was not required considering the nature of the study.

We searched, without language restriction, MEDLINE, EMBASE, and the Cochrane Library through December 2015. The search terms were ischemic preconditioning, RIPC, cardiac surgical procedures, cardiac surgery, coronary artery bypass graft surgery, valve surgery, CPB, and RCTs. Reference lists of retrieved articles were manually searched to avoid omissions.

### Types of Outcome Measures

The primary outcomes were development of AKI, initiation of RRT. Serum creatinine (sCr) levels and in-hospital mortality after surgery were secondary outcomes.

### Study Selection

The inclusion criteria were as follows: patients underwent on-pump cardiac surgical procedures, including coronary artery bypass graft surgery and valve surgery; and studies comparing RIPC with control, and sufficient data available to calculate a relative ratio (RR) or mean difference (MD) with 95% confidence interval (95% CI). The following exclusion criteria were used: RIPC was performed in all patients undergoing major vessel surgery; study protocols; pediatric patients; nonhuman studies; and percutaneous coronary interventions/coronary angiography studies.

Two investigators (YZ and XZ) independently reviewed all abstracts and included the full-text each trial independently and recorded eligibility, quality, and outcomes. Disagreements between the reviewers concerning the decision to include or exclude a study were resolved through discussion. If necessary, the 3rd reviewer (DC) was consulted. We excluded duplicate reports, non-RCTs, and experimental design. Conference abstracts were also excluded, unless published as full-text reports in journals.

### Quality Assessment

Two reviewers (YZ and XZ) independently performed quality assessment. We used the Jadad scoring system to assess the quality of the trails according to randomization; blinding; withdrawals; and dropouts.^17^ According to this scale, we judged the trails as low-quality study with 2 or less points and high-quality study with 3 or more points.

### Data Synthesis and Analysis

Before the analysis, data were standardized into equivalent units. We calculated and subsequently pooled in independent meta-analyses, risk ratio (RR) with 95% CI for dichotomous outcomes and MD with 95% CI for continuous outcomes. Heterogeneity among pooled studies was evaluated using the Mantel–Haenszel, χ^2^, and the I^2^ statistic to assess the degree of interstudy variation. I^2^ values exceeding 25%, 50%, and 75% were considered evidence of low, moderate, and severe statistical heterogeneity, respectively.^18^ Homogeneity assumption was measured by *P* value. A *P* value <0.10 indicates statistically significant heterogeneity and synthesis of each study was performed using the random-effects model.

Publication bias was evaluated by Begg test and Egger test. Sensitivity analysis was conducted by sequentially deleting a single study each time in an attempt to identify the potential influence of an individual study. A 2-tailed *P* value of <0.05 was considered a criterion for statistical significance. Analyses were carried out with Review Manager 5.3 (RevMan, The Cochrane Collaboration, Oxford, UK) and STATA 12.0 (StataCorp, College Station, TX).

## RESULTS

### Study Characteristics

**FIGURE 1:**
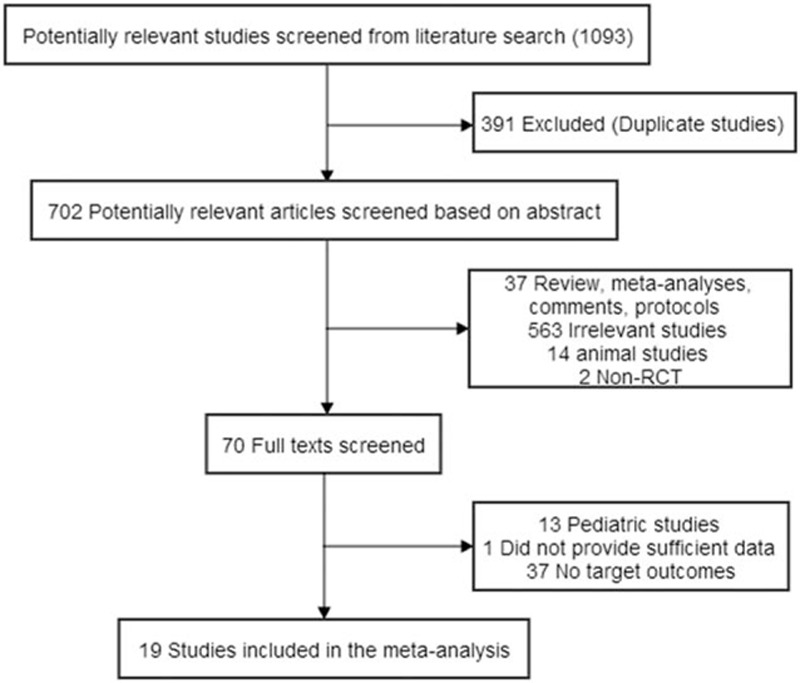
Flow chart of selection process of eligible studies. RCT = randomized controlled trial.

The study selection process is presented in Figure 1. The search strategy identified 1093 studies, of which data from 19 trials^19–37^ were used comprising 5100 patients (Table 1). Among the 19 studies, 1 was a conference abstract,^21^ which has not been published as full-text reports in a peer-reviewed journal. One study by Walsh and his colleagues included 7 patients (accounting 2.7%) with off-pump procedure.^37^ Two studies^19,21^ had a Jadad score of <3 (Table 2). For the primary endpoints, the incidence of AKI was reported in 14 trails.^20–25,28–30,32–36^ In this part, the multicenter randomized study by Meybohm et al^27^ was not analyzed, because the patient of AKI grade 1 were not recorded according their supplementary materials. The incidence of renal-replacement therapy was reported in 9 trails.^19,23,26,27,29–32,35^

**TABLE 1:**
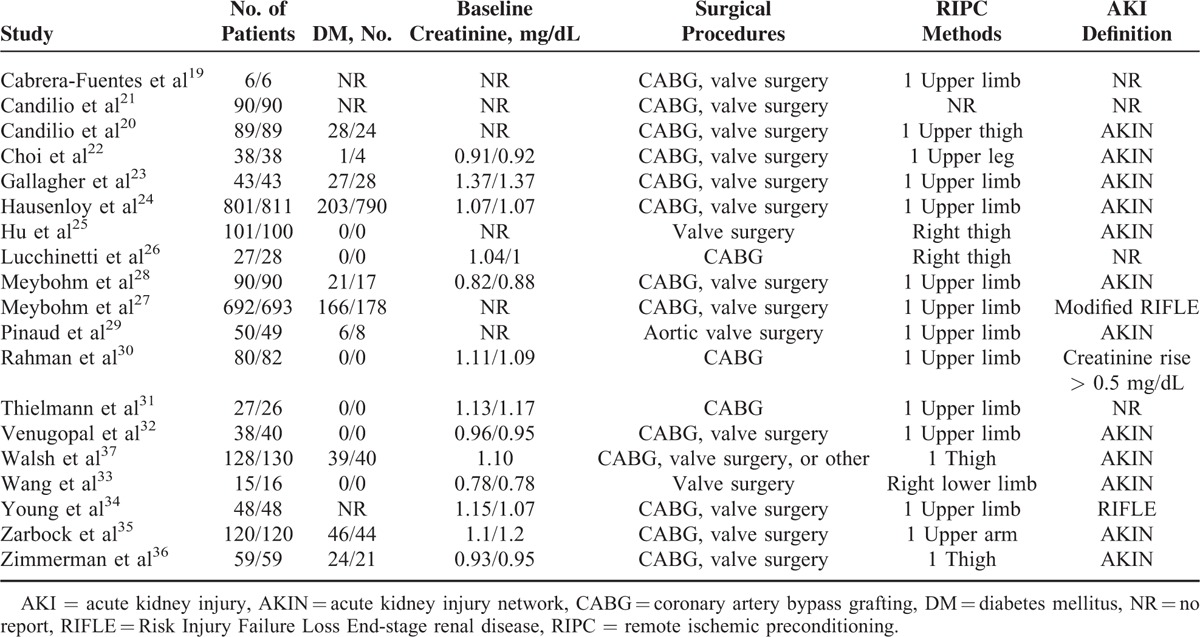
Demographic Data of Studies Included in Meta-Analysis (RIPC Group/Control Group)

**TABLE 2:**
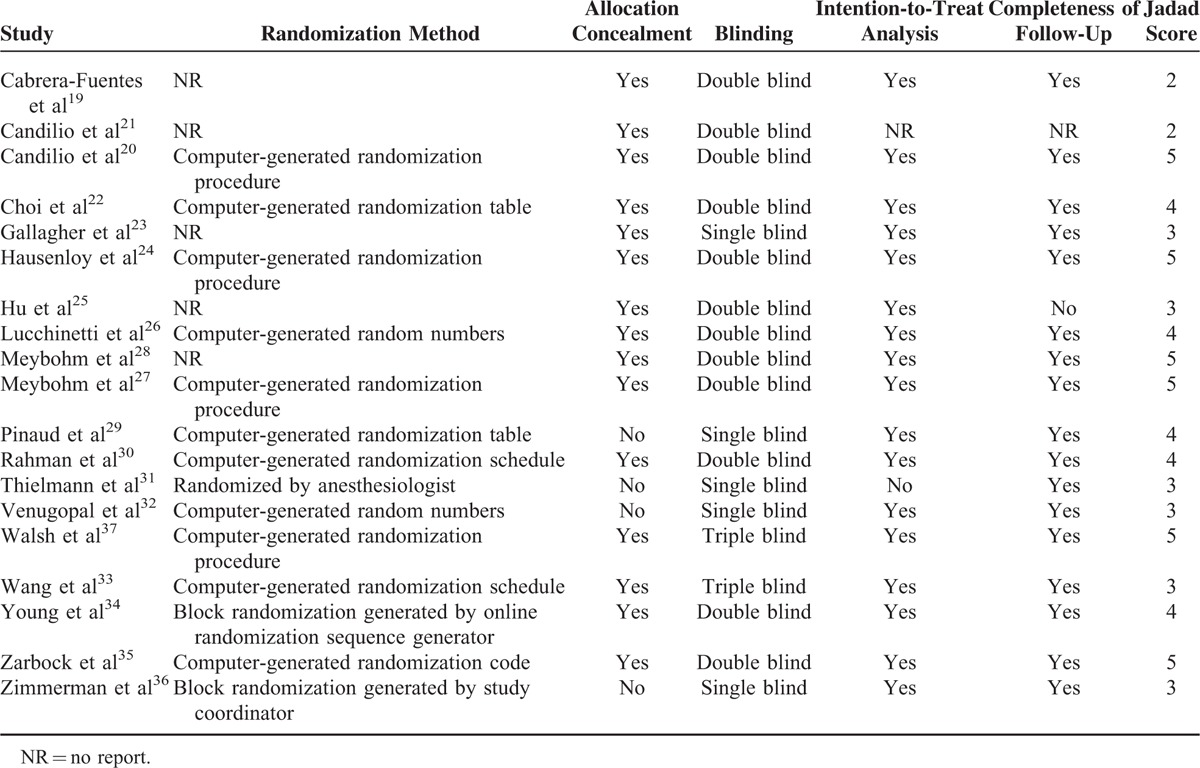
Quality of Studies Included in Meta-Analysis

### Effect of RIPC on the Incidence of AKI

**FIGURE 2:**
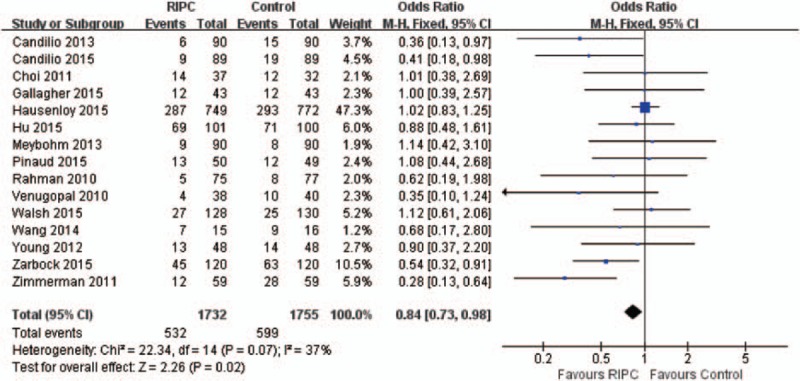
Forest plot for acute kidney injury.

A total of 15 RCTs including 3487 patients reported data on the incidence of AKI, and the overall incidence was 32.4% (532/1732 in RIPC group and 599/1755 in control group). There was a significantly lower risk of AKI in the RIPC group compared with control group using the fixed-effect model (odds ratio [OR] = 0.84; 95% CI, 0.73–0.98; *P* = 0.02), with low heterogeneity (χ^2^ = 22.34, I^2^ = 37%) (Figure 2). Sensitivity analysis sequentially deleting a single study each time revealed that most individual study was consisted. The RR did not change markedly in sensitivity analyses, ranging from 0.69 (0.51, 0.81) with lower heterogeneity (I^2^ = 20%) when the trial by Hausenloy et al^24^ was omitted to 0.88 (0.75, 1.02) (I^2^ = 17%) when the trial by Zimmerman et al.^36^ No significant publication bias was detected, with *P* = 0.843 in Begg test and *P* = 0.055 in Egger test.

### Effect of RIPC on RRT

**FIGURE 3:**
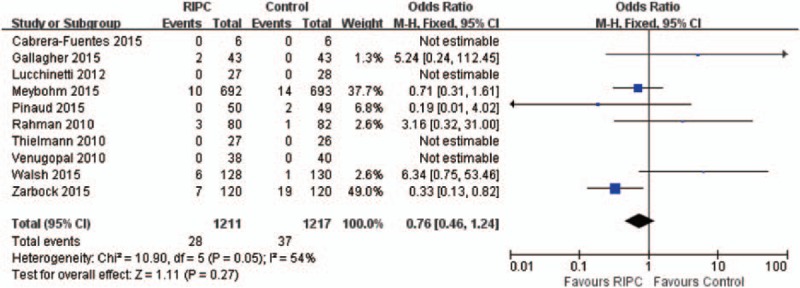
Forest plot for renal replacement therapy.

The initiation of RRT was reported in 2428 study subjects. In 4 of the 10 trials, none of patients required postoperative hemodialysis or hemofiltration. In total, 65 patients, 2.68%, received RRT after surgery (28, RIPC group; 37, control group). There was no significant difference in the incidence of RRT between 2 groups (OR, 0.76, 95% CI, 0.46–1.24; *P* = 0.27) with nonsignificant heterogeneity (χ^2^ = 10.90, I^2^ = 54%) (Figure 3). In sensitivity analysis, there was no significant difference between 2 groups for replacement therapy requirement. No evidence of publication bias was detected for initiation of RRT by either funnel plots, Begg test (*P* = 1.000), or Egger test (*P* = 0.237).

### Postoperative sCr Levels

**FIGURE 4:**
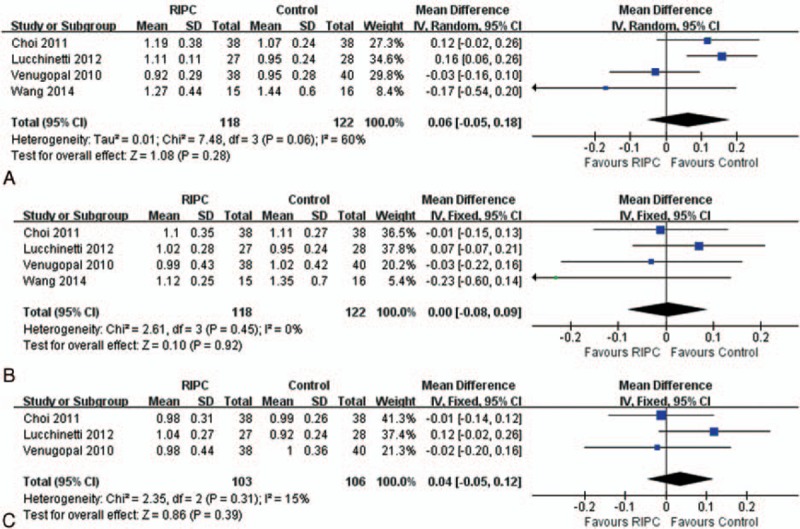
Forest plot for postoperative serum creatinine levels. (A) Serum creatinine levels on the 1st postoperative day; (B) serum creatinine levels on the 2nd postoperative day; and (C) serum creatinine levels on the 3rd postoperative day.

Available information on the hospital postoperative sCr was analyzed. No statistically significant difference was observed on 1, 2, or 3 days after operation (Figure 4).

### Mortality

**FIGURE 5:**
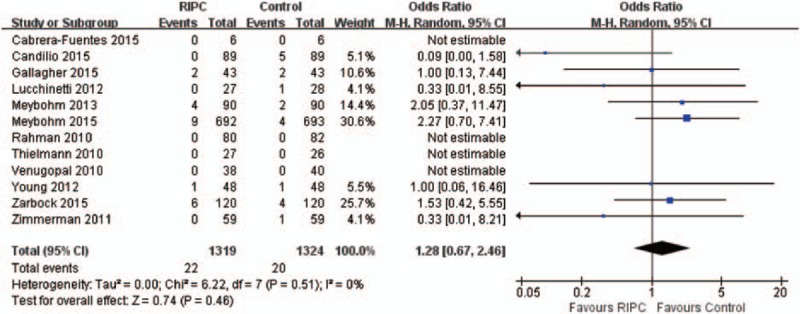
Forest plot for relative risk of mortality.

A total of 13 studies including 2710 patients reported data on the in-hospital mortality. There was no statistically significant difference in the overall mortality between 2 groups (OR, 1.21, 95% CI, 0.64–2.30; *P* = 0.56) with no heterogeneity (χ^2^ = 6.97, I^2^ = 0%) (Figure 5).

## DISCUSSION

This study reports detailed analyses of 19 trails that compared RIPC to the control group in prevention of AKI for patients undergoing cardiac interventions. The results of this meta-analysis showed a significant benefit of RIPC for reducing the incidence of AKI after cardiac interventions. No significant differences were found in the incidence of RRT, postoperative sCr, and in-hospital mortality.

AKI is a potential complication among patients undergoing cardiac surgery that can arise from a variety of causes. Data suggest that even a small increase (0.3–0.5 mg/dL) in sCr after cardiac surgery is associated with a substantial decrease in survival.^38^ The protection was supported by numerous preclinical studies in animals showing that RIPC, compared with controls, was associated with reduction of blood urea nitrogen (BUN), sCr, and histologic renal damage.^39,40^

The present analysis is in accordance with the findings of a previous meta-analysis, and helps further understand the effect of RIPC.^41^ In addition to experimental evidence, several clinical trials have suggested the potential protection of RIPC on reducing kidney damage in humans,^20,36^ while some studies found the contrary.^25,29^ Lack of knowledge of RIPC and conflicting results of small sample sizes trials^20,21,30,33^ may explain the absence of RIPC in the operating room. The inconsistent outcomes across RIPC trials might also have been due to differences in study protocols, different patient populations, comorbid diseases, anesthetic regimens, and surgical technique.^40^ To achieve widespread clinical acceptance of RIPC, it has been suggested that focus should be kept on patients at high risk of global tissue damage including AKI.^42^ They might benefit most from protection by RIPC.

A prospective double-blind study performed by Zarbock et al reported that patients undergoing cardiac surgery were at high risk of AKI with a Cleveland Clinic score ≥6. Their study included 240 subjects in which the rate and severity of AKI influenced by RIPC was compared with a sham procedure.^35^ They found fewer patients in the RIPC group received RRT (5.8% vs 15.8%, respectively) and shorter intensive care unit stay length than our study. As for there was no significance on the secondary end points including of in-hospital, and 30-day mortality, mechanical ventilation, myocardial infarction, or stroke no significant difference was detected.

However, the result should be interpreted with caution. The definitions of AKI adopted in respective trials included in the present meta-analysis were different which might influence the global results. The adopted definitions included the AKIN criterion, RIFLE criterion, and other similar definitions. Although all the adopted AKI definitions were similar, the conclusions drawn should be treated with caution. However, the subgroup analysis based on definitions of AKI of a previous meta-analysis indicated a nonsignificant effect of RIPC on AKI prevention.^41^

The absence of an association between RIPC and RRT could be expected, since requirement of replacement therapy is very low in general, and only 2.68% patients developed into a severe dialysis-dependent AKI in our present analysis.

### Limitations

The present meta-analysis has several limitations that should be considered. First, the definitions of AKI adopted in respective trials were different, although no significant effect was found. Second, comorbidities among the studies were different such as hyperlipidemia, diabetes, and hypertension. They may raise protective threshold and to some extent resist to various conditioning strategies. In our analysis, the heterogeneity is low and acceptable. Third, preoperative kidney function of the studies was different, and patients at high risk of AKI following cardiac surgery were supposed to benefit from RIPC most. We lacked corresponding trails to make a subgroup analysis and whether preexisting decreased kidney function could influence the effect of RIPC on AKI. Fourth, different anesthetic protocols might confound the effect of RIPC. Both propofol anesthesia and volatile anesthetics were shown to attenuate protection by RIPC.^27,43^ Opioid analgesics can interfere with cardioprotective efficacy of RIPC and raise the threshold for an additional benefit by their independently cardioprotection.^42^

## CONCLUSION

In present systematic meta-analysis, RIPC was associated with a significant reduction AKI after on-pump cardiac surgery but incidence of RRT, postoperative sCr, and in-hospital mortality. Numerous clinical trials using several interventions to prevent AKI have been somewhat disappointing. In clinical practice, RIPC appears to be a safe procedure, as no adverse events related to RIPC application were reported to date.

To verify the beneficial effects of RIPC, more efforts should be made to form a better evidentiary basis for RIPC. Before RIPC is adopted for clinical use, large-scale, probably multicenter, and high-quality studies will be needed to change practice. Meanwhile more experimental research is needed on the potential mechanisms responsible for improved AKI.
